# Effects of a mindfulness intervention on emotion differentiation and heart rate variability

**DOI:** 10.3389/fnhum.2025.1515334

**Published:** 2025-07-02

**Authors:** Simón Guendelman, Martina Lutz, Julian Koenig, Mareike Bayer, Isabel Dziobek

**Affiliations:** ^1^Berlin School of Mind and Brain, Humboldt-Universität zu Berlin, Berlin, Germany; ^2^Clinical Psychology of Social Interaction, Institute of Psychology, Humboldt-Universität zu Berlin, Berlin, Germany; ^3^Department of Child and Adolescent Psychiatry, Psychosomatics and Psychotherapy, Faculty of Medicine and University Hospital Cologne, University of Cologne, Cologne, Germany; ^4^German Center for Mental Health (DZPG), Partner Site Berlin-Potsdam, Berlin, Germany

**Keywords:** mindfulness, emotion differentiation, heart rate variability, top-down, bottom-up, emotion regulation, mechanisms

## Abstract

This study explored the impact of a mindfulness-based intervention (MBI) on mental health and emotion regulation mechanisms, particularly focusing on the top-down and bottom-up related processes, such as emotion differentiation and heart rate variability (HRV). Participants underwent an 8-week mindfulness-based stress reduction (MBSR) program or a reading-sharing intervention (READ). Emotion differentiation and HRV were measured at various intervals, alongside mental health measurements (depressive and stress symptomatology, wellbeing, mindfulness, and self-compassion traits). The results showed that the MBSR group exhibited increased emotion differentiation for negative emotions and improvements in various mental health measurements (wellbeing and mindfulness traits) compared to the READ group. However, there were no significant differences in HRV between the two groups, and gains in emotion differentiation did not correlate significantly with changes in mental health outcomes. The findings suggest that MBIs can enhance emotion differentiation as a top-down mechanism and improve mental health outcomes. However, further research is needed to understand the precise psychophysiological mechanisms underlying these effects.

## Introduction

In recent years, mindfulness-based interventions (MBIs) have gained extensive popularity as mental health therapies. MBIs are usually defined as group-based, manualized, time-limited interventions that foster mindfulness through different practices including meditation, yoga, and daily life exercises (Shonin et al., 2013). In this context, mindfulness has been defined as the capacity to be aware of the present moment experience with a “non-elaborative and non-judgmental attitude” (Kabat-Zinn, 2005). Although various studies and meta-analyses have demonstrated that MBIs can both increase positive emotions and wellbeing (van Agteren et al., 2021) and decrease negative emotions and psychopathology (Goldberg et al., 2022), there is limited understanding of the underlying emotion-related and neurophysiological mechanisms.

Emotion regulation, i.e., the implementation of the different strategies we use to modify our current emotional experience (Gross, 1998), has been identified as a potential core mechanism involved in MBIs’ salutary effects (Tang et al., 2015; Guendelman et al., 2017). Regarding its neurobiological underpinnings, several studies have revealed the involvement of different high-level (cognitive), mid-level (affect), and low-level (sensory) brain networks in the orchestration of emotion regulation (Etkin et al., 2015; Brandl et al., 2018). From this perspective, emotion regulation may rely on the interaction of different top-down (mainly high-level cognitive control) and bottom-up (mainly affective and sensory-interoceptive processing levels) neurophysiological processes (Guendelman et al., 2017; Bramson et al., 2023).

More specifically, top-down regulation refers to the emotion regulation strategies that modulate the cognitive input to the affective and sensory levels (emotion generation) and include strategies such as reinterpretation and reappraisal of emotional experiences. These strategies involve prefrontal cortices brain regions, and higher-level cognitive processes (Chiesa et al., 2013). Emotion differentiation (ED), which refers to the ability to distinguish between one’s own similar yet different emotions, instead of merely categorizing them into “good” or “bad” (Lindquist and Barrett, 2008), can be considered a top-down strategy as it manipulates the cognitive input to the emotion-generative system by actively interpreting the current emotional state in a differentiated fashion. Studies have shown that individuals with higher ED abilities experience less negative and more positive emotions and wellbeing as well as better social adjustment across clinical and non-clinical populations (Smidt and Suvak, 2015). ED has been associated with reduced use of dysfunctional emotion regulation strategies such as binge drinking, aggression, and self-injurious behavior (Kashdan et al., 2015). In the context of MBIs, participants are encouraged to recognize and distinguish emotional states in a differentiated manner. Interestingly, in the mindfulness literature, a cross-sectional study showed that dispositional mindfulness was positively associated with ED and that the latter predicted lower emotional reactivity (Hill and Updegraff, 2012). Furthermore, a longitudinal study investigated ED in the context of MBIs: using a pre-post single group design, participants in the mindfulness-based stress reduction (MBSR) program showed an increase in ED of both negative and positive emotions after the intervention (Van Der Gucht et al., 2019). This finding, although preliminary, suggests that MBIs can enhance ED, possibly reflecting improved emotion regulation.

Concomitantly, bottom-up regulation can be conceived as encompassing strategies that can directly influence affective and sensory processing. These strategies can be understood as experiential engagements or concrete, action-oriented responses. Rather than involving cognitive reframing and control processes, they rely on the direct modulation of emotion-generating brain regions (e.g., the amygdala or insular cortex) (Chiesa et al., 2013; Guendelman et al., 2017) and may include focusing on physical sensations (e.g., using soothing touch) or exposure to emotional experiences (e.g., through emotional content exposure). It has been stated that bottom-up strategies facilitate emotion regulation by directly influencing the negative valence system (Raugh and Strauss, 2024), targeting circuits related to rewards and interoceptions (Kirk et al., 2015), and possibly relying on emotion reinforcement learning mechanisms—i.e., modifying the encoding of negatively valenced experiences (Sevinc et al., 2019).

Heart rate variability (HRV), i.e., the degree of variation between heartbeat intervals, is conceived as an index of the autonomic parasympathetic nervous system (influencing visceral functions such as heart and respiratory rates) (Thayer et al., 2011). HRV can be considered a key element in bottom-up emotion regulation as it indexes emotion generation (arousal) and somatosensory brain processing (autonomic and cardiac signals) (Smith et al., 2017; Mather and Thayer, 2018). Many studies have associated emotion regulation with HRV, indicating that subjects with higher resting HRV (as measured during rest) are better able to regulate negative emotions and more often use adaptive and more flexible emotion regulation strategies (Balzarotti et al., 2017). Intriguingly, in the context of mindfulness studies, previous meta-analyses investigating non-randomized and randomized controlled studies have shown no conclusive effects of MBI on resting HRV (Rådmark et al., 2019; Brown et al., 2021).

Even though previous studies have shown top-down and bottom-up processes as relevant mechanisms for emotion regulation in MBIs (Chiesa et al., 2013; Guendelman et al., 2017), to the best of our knowledge, no previous studies have investigated ED specifically in the context of an MBSR active-controlled randomized trial. Furthermore, no previous research has concomitantly investigated ED and HRV, thus testing both top-down and bottom-up processes. In this study, we tested whether the emotion regulation mechanisms behind MBSR involve top-down mechanism, as indexed by increased ED, or a bottom-up mechanism, i.e., a direct influence on the bodily stress system, as indexed by increased HRV. We also tested the effects of MBSR on standard mental health outcomes, including depressive (Beck Depression Inventory) and stress (Perceived Stress Scale) symptomatology, wellbeing (Flourishing Scale), self-compassion (Self-Compassion Scale), and mindfulness (Freiburg Mindfulness Inventory) traits. Furthermore, and more exploratorily, we investigated whether ED and HRV were actively involved in outcome benefits through their association with mental health outcome measurements.

## Methods

### Subjects and recruitment

A total of 550 participants were screened from the general population (in the city of Berlin, Germany), and 68 healthy participants with no previous meditation experience who met the inclusion criteria were randomly assigned to either the experimental (MBSR, *n* = 34) or the control (READ, *n* = 34) group. Groups did not differ in gender, age, or education (See Supplementary Table 1). A stratified procedure was used to randomly assign subjects, ensuring an equal number of male participants in each group, to secure gender balance in both arms of the trial. A higher rate of female participants is a well-known selection bias in MBI trials (Guendelman et al., 2022).

The study was preregistered at clinicaltrials.gov (id# NCT03035669); the main aim was to investigate functional brain mechanisms as the primary outcome measure of the trial. These data were already published (Guendelman et al., 2022). In the present article, we report the results from a questionnaire targeting emotion differentiation, which were applied throughout the study, and HRV and mental health questionnaires, which were measured pre- and post-intervention.

### Interventions

In this randomized controlled trial (RCT), participants were assigned to either an 8-week mindfulness-based stress reduction (MBSR) intervention (target intervention group) or a reading-sharing intervention (READ) (active control group). Both were matched in terms of weekly meetings and home assignments for a more detailed description of both interventions (see the previous publication) (Guendelman et al., 2022).

### Assessments

#### Self-report questionnaires

*Depressive symptomatology* was measured using the 21-item Beck Depression Inventory (BDI) (Beck et al., 1988), in its German version (Hautzinger et al., 1994). Subjects self-respond on a three-point Likert scale from 0 (never) to 3 (very often).

*Perceived stress* was estimated using the 10-item Perceived Stress Scale (PSS) (Cohen, 1994) in its German version (Klein et al., 2016). Participants responded on a five-point Likert scale from 0 (never) to 4 (very often).

*Wellbeing* was measured with the 8-item Flourishing Scale (FSD) (Diener et al., 2009) in its German version (Esch et al., 2013), and subjects responded on a seven-point Likert scale from 1 (not agree at all) to 7 (very agree).

*Mindfulness traits* were measured with the 14-item Freiburg Mindfulness Inventory (FMI) (Walach et al., 2006); participants respond on a four-point Likert scale from 1 (rarely) to 4 (almost always).

*Self-compassion* was measured with the 26-item Self-Compassion Scale (SCS) (Neff, 2003) in its German version (Hupfeld and Ruffieux, 2011). The SCS consists of three bipolar subscales; participants responded on a five-point Likert scale from 1 (almost never) to 5 (almost always).

### Statistical analyses

Self-report questionnaires (as mental health outcomes) data were analyzed with a 2 (pre vs. post-intervention) × 2 (group: MBSR vs. READ) repeated measures ANOVA, followed by *post-hoc* pairwise comparisons with Holm’s corrections.

To elucidate the relationship between emotion differentiation trajectory changes and mental health outcomes, a correlational analysis (using two-tailed *Pearson’s r* correlations) using emotion differentiation trajectory (GLMM estimates) and questionnaire change score (T2 – T1) was performed. Analyses were performed with JASP, version 0.9.1, University of Amsterdam.

### Emotion differentiation (ED)

ED was measured using the Modified Differential Emotions Scale (mDES) (Fredrickson, 2013), using the German version (Brandenburg and Backhaus, 2015). The questionnaire was administered through an online portal and consisted of 20 items. Each item targeted a specific emotion, with 10 of the items targeting positive and 10 targeting negative emotions. Participants rated the degree to which they have experienced each of the 10 positive and 10 negative emotions using a five-point Likert scale ranging from zero (“not at all”) to four (“very strongly”). The mDES was measured approximately every 4 days at up to 13 different time points throughout the study, including pre- and post-intervention measures.

To estimate ED, intraclass correlation coefficients (ICCs) (Shrout and Fleiss, 1979) for mDES responses were calculated. As in reliability studies, ICC estimates the relatedness or consistency of the ratings; in this case, for the 10 different but like-valenced emotions, ICCs (type 3) were calculated separately for positive and negative emotions. When inverting the ICC score, the result indicated the non-relatedness, or, in other words, the distinctiveness of the 10 like-valenced emotions, i.e., subjects’ ability to differentiate between the 10 like-valenced emotions. As suggested by Anand et al. (2017), a minimum number of responses are needed to estimate reliable and stable ICC scores; therefore, the 13 time points were consolidated into four time periods that indexed the trajectory of mDES responses throughout the interventions. The ICC scores were calculated through the R package “psych” version 2.0.8 (Revelle, 2020).

### Heart rate variability (HRV)

Heart rate was collected with a Biopac MP-150 system with a sampling rate of 100 Hz. A photo-plethysmography pulse detector device was placed on the left middle finger. Heart rate peaks were detected using the automatic multiscale-based peak algorithm (Scholkmann et al., 2012). Inter-peak distances were analyzed using Kubios HRV Premium version 3.1.0. Resting HRV was estimated from the heart rate data acquired during the scanner experiment, specifically during the 5 min of structural brain acquisition period. Resting HRV was measured at two time points, before the start of the intervention and after finishing the 8 weeks of intervention, estimating both the root mean square of successive RR interval differences (RMSSD) and the power of the high-frequency band of HRV (HF-HRV).

### Statistical analyses

As for ED, a linear mixed-effects model (LMM) approach was used for analyzing the data, with a *group* (MBSR vs. READ) by *time* (ICC with four time periods) interaction as the main fixed effect, as well as for the random effects, controlling for intra-subject variation. Given the non-normal distribution of the residuals, a generalized linear mixed-effects model (GLMM) approach was utilized using a Gamma distribution. The data analyses were performed in R version 4.0.2. (R Core Team, 2020), using the R package “lmerTest” version 3.1-3 (Kuznetsova et al., 2017). Regression tables and figures displaying the results of the GLMMs were produced using the R packages “effects” version 4.2-0 (Fox and Weisberg, 2019), “sj-plot” version 2.8.6 (Lüdecke, 2020), and “ggplot2” version 3.3.2 (Wickham, 2016).

In case of HRV, a linear mixed-effects model (LMM) approach was used for analyzing the data, with a *group* (MBSR vs. READ) by *time* (pre- and post-intervention) interaction as the main fixed effect, as well as for the random effects, controlling for intra-subject variation. Analyses were performed with JASP, version 0.9.1, University of Amsterdam.

## Results

### Effects of MBSR versus READ on negative emotion differentiation time change

**Figure 1:**
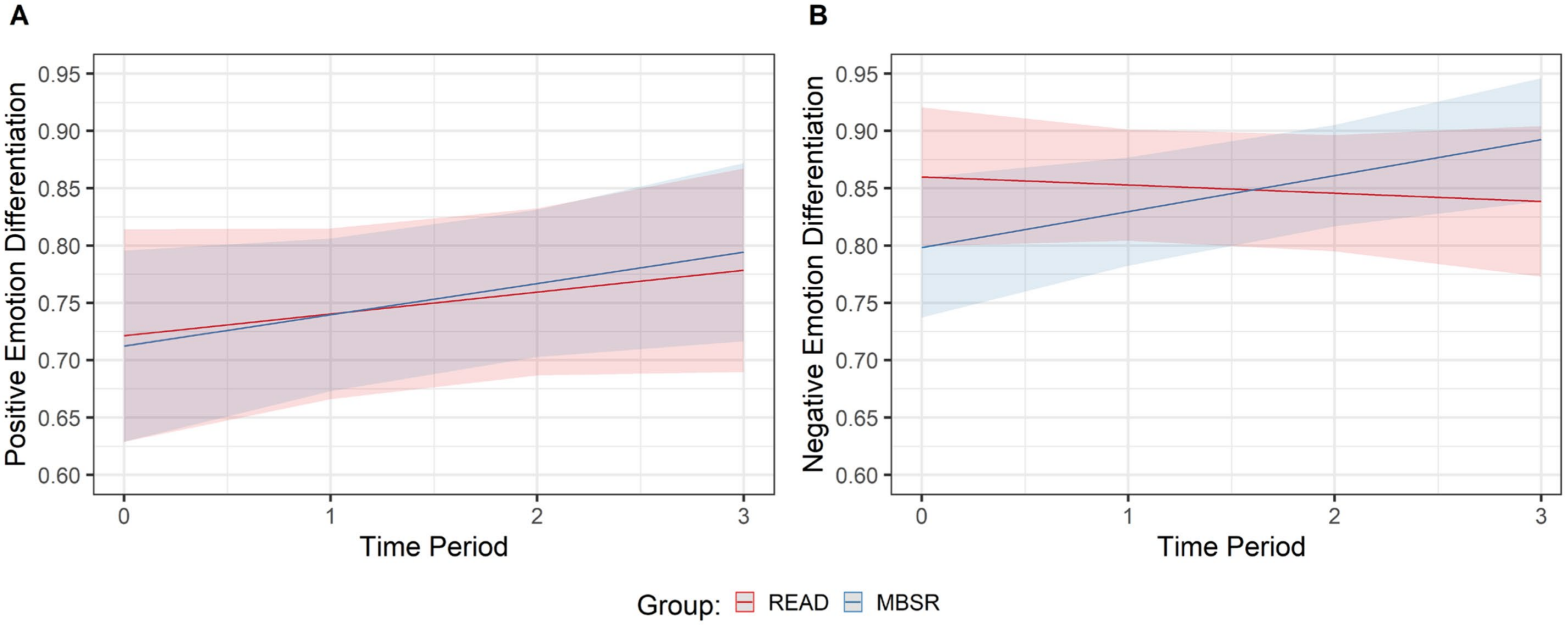
Positive and negative emotion differentiation over time by group. Modeled trajectories of **(A)** positive and **(B)** negative emotion differentiation over time for the mindfulness-based stress reduction (MBSR) and the active control group (READ), respectively. Shadowed bands represent 95% confidence intervals.

For negative emotion differentiation, the GLMM model resulted in a significant group by time interaction (*X*^2^_1_ = 4.21, *p* = 0.04), demonstrating that the trajectory of the MBSR group was estimated to be more positive than that of the READ group by +0.04 points, indicating a medium effect size (Table 1 and Figure 1).

**Table 1:** Generalized linear mixed-effects model analyses: effects of group and time on subjects’ positive and negative emotion differentiation.

Effect	Positive emotion differentiation					Negative emotion differentiation				
	Estimate	*SE*	95% CI		*p*	Estimate	*SE*	95% CI		*p*
			*LL*	*UL*				*LL*	*UL*	
Fixed effects										
Intercept^a^	0.72	0.05	0.63	0.81	<0.001	0.86	0.03	0.80	0.92	<0.001
Group^b^	−0.01	0.06	−0.13	0.11	0.884	−0.06	0.04	−0.15	−0.02	0.153
Time^c^	0.02	0.02	−0.02	0.06	0.316	−0.01	0.01	−0.03	0.02	0.611
Group * Time^d^	0.01	0.03	−0.04	0.06	0.744	0.04	0.02	0.00	0.08	0.040
Random effects										
Within-subject variance	0.40					0.45				
Between-subject variance	0.01					0.01				

Number of subjects = 54; observations for positive emotion differentiation = 201; observations for negative emotion differentiation = 198. CI = confidence interval; LL = lower limit; UL = upper limit. Regression results were derived from generalized linear mixed-effects models (GLMMs) modeling intraclass correlation coefficients (ICCs) of subjects’ positive and negative emotion ratings over time, with subjects belonging either to the mindfulness-based intervention group (MBSR) or an active control group (READ). The results displayed in the table were transformed to readily index emotion differentiation. As the GLMMs were based on treatment contrasts and included an interaction term for the predictor variables time and group, the estimated intercepts and slopes are given for the baseline condition, i.e., the READ group, and are complemented by estimates quantifying the MBSR group’s deviations from the READ group’s intercepts and slopes.

a Intercept estimated for the READ group: level of the READ group’s (positive or negative) emotion differentiation at the beginning of the intervention.

b Estimated difference between the intercepts of the MBSR group and the READ group: degree to which the MBSR group’s intercept differs from the READ group’s intercept.

c Slope estimated for the READ group: rate of change of the READ group’s (positive or negative) emotion differentiation.

d Estimated difference between the slopes of the MBSR group and the READ group: degree to which the MBSR group’s slope differs from the READ group’s slope.

### Effects of MBSR versus READ on positive emotion differentiation time change

For positive emotion differentiation, the GLMM model resulted in a non-significant group by time interaction (*X*^2^_1_ = 0.11, *p* = 0.75), demonstrating that the trajectories of both MBSR and READ groups did not differ from each other (Table 1 and Figure 1).

### Effects of MBSR versus READ on resting heart rate variability

For HRV measurements (RMSSD and HF-HRV), the LMM model did not result in a significant group by time interaction for RMSSD (*F*_1,46_ = 0.22, *p* = 0.64), nor for HF-HRV (*F*_1,46_ = 0.78, *p* = 0.40), suggesting that, compared to READ, the MBSR group did not increase resting HRV over time (Table 2).

**Table 2:** MBSR and READ groups mean (M) and standard deviation (SD) values for resting HRV estimates and mental health outcomes, both pre- and post-intervention assessments.

Outcome	MBSR Pre- M (SD)	MBSR Post- M (SD)	READ Pre- M (SD)	READ Post- M (SD)	ANOVAS *F*-tests and (*p*-values)
Resting HRV (RMSSD)	36.8 (19)	40.7 (17)	41.8 (22)	43.4 (20)	*F*_1,46_ = 0.22 (*p* = 0.64)
Resting HRV (MS^2^)	622 (740)	779 (560)	924 (837)	901 (838)	*F*_1,46_ = 0.78 (*p* = 0.40)
Depressive symptomatology (BDI)	10.3 (7.2)	6.4 (5.3)	9.5 (6.4)	7.6 (4.3)	*F*_1,50_ = 2.1 (*p* = 0.15)
Perceived Stress Scale (PSS)	17.6 (7.2)	14.2 (5.4)	17.8 (7.0)	15.8 (7.6)	*F*_1,50_ = 1.3 (*p* = 0.25)
Wellbeing (FSD)	45.3 (5.9)	47.9 (5.8)	46 (4.2)	46.1 (6.2)	*F*_1,50_ = 4.9 (*p* = 0.03)
Mindfulness trait (FMI)	2.52 (0.5)	2.89 (0.3)	2.62 (0.5)	2.64 (0.5)	*F*_1,50_ = 8.2 (*p* = 0.006)
Self-compassion (SCS)	3.18 (0.7)	3.54 (0.5)	3.23 (0.6)	3.33 (0.6)	*F*_1,50_ = 3.4 (*p* = 0.07)

RMSSD, root mean squared of standard deviation; MS^2^, squared milliseconds; BDI, Beck Depression Inventory; PSS, Perceived Stress Scale; FSD, Flourishing Scale Deutsch; FMI, Freiburg Mindfulness Inventory; SCS, Self-Compassion Scale.

### Effects of MBSR versus READ on mental health outcome (self-reported questionnaires)

For depressive symptomatology, the repeated measures ANOVA resulted in a non-significant group by time interaction (*F*_1,50_ = 2.1, *p* = 0.15, *η*^2^_p_ = 0.04); however, a significant main effect of time suggests that both groups experienced decreased depressive symptomatology over time (*F*_1,50_ = 17.7, *p* < 0.001, *η*^2^_p_ = 0.26). For perceived stress, the repeated measures ANOVA resulted in a non-significant group by time interaction (*F*_1,50_ = 1.3, *p* = 0.25, *η*^2^_p_ = 0.03); however, a significant main effect of time suggests that both groups experienced decreased perceived stress over time (*F*_1,50_ = 7.3, *p* = 0.01, *η*^2^_p_ = 0.13) (Table 2).

For wellbeing, the repeated measures ANOVA resulted in a significant group by time interaction (*F*_1,50_ = 4.9, *p* = 0.03, *η*^2^_p_ = 0.09), demonstrating that, compared to the READ group, the MBSR group experienced an increase in perceived wellbeing over time (Holms corrected-*p* = 0.02). For mindfulness traits, the repeated measures ANOVA resulted in a significant group by time interaction (*F*_1,50_ = 8.2, *p* = 0.006, *η*^2^_p_ = 0.14), demonstrating that, compared to READ, the MBSR group experienced an increase in mindfulness traits over time (Holms corrected-*p* < 0.001). For self-compassion, the repeated measures ANOVA resulted in a non-significant group by time interaction (*F*_1,50_ = 3.4, *p* = 0.07, *η*^2^_p_ = 0.06); however, a significant main effect of time suggests that both groups experienced increased self-compassion over time (*F*_1,50_ = 10.9, *p* = 0.002, *η*^2^_p_ = 0.18) (Table 2).

### Associations between emotion differentiation time change and mental health outcome change scores

The correlational analyses examined the relationship between increases in negative ED over time and change scores on mental health questionnaires, which indicated non-significant associations between negative ED gains and changes over time in depression (*r* = −0.11, *p* = 0.45), perceived stress (*r* = −0.13, *p* = 0.39), wellbeing (*r* = 0.26, *p* = 0.086), mindfulness (*r* = 0.29, *p* = 0.085), and self-compassion traits (*r* = 0.11, *p* = 0.46).

## Discussion

The present active-controlled randomized study investigated the impact of the MBSR on mental health and emotion regulation psychophysiological mechanisms, focusing on ED and HRV as top-down and bottom-up related processes, respectively. Our results indicated that both groups experienced mental health benefits (as indexed by decreased depressive and stress symptomatology), but only the MBSR group demonstrated improved wellbeing and mindfulness traits compared to the READ group. The MBSR group increased ED capacity for negative, but not positive, emotions. Nevertheless, there were no significant changes in HRV when comparing both groups, and changes in negative and positive ED did not correlate significantly with mental health outcomes. Our findings suggest that MBSR can enhance negative ED as a top-down mechanism, albeit not a bottom-up mechanism, as increases in HRV could not be demonstrated.

### Effects of MBSR on mental health outcomes

The study resulted in non-significant group differences between MBSR and READ interventions, specifically regarding depressive and stress symptomatology. As in previous studies implementing active control interventions, both groups exhibited decreases in depressive and stress symptoms (Goldberg et al., 2022). The decreases could be due to non-specific effects provided by the READ intervention, such as group support, weekly sessions, and daily basis home assignments. However, only the MBSR showed increased wellbeing and mindfulness trait, suggestive of a more specific effect of MBSR, i.e., as active mechanisms might be in place, including the learning of emotion regulation strategies such as cognitive reappraisal and mindful acceptance (Goldin et al., 2021; Guendelman et al., 2022), in addition to emotion differentiation.

### Effects of MBSR on emotion differentiation

To the best of our knowledge, this is the first longitudinal study investigating ED in the context of MBI using an RCT design. Our findings are in line with a previous pre-post single-group study, which demonstrated that MBSR can increase ED for both positive and negative emotions (Van Der Gucht et al., 2019). Unlike this study, our study only showed effects for ED for negative emotions. This could be due to the trial design, since our study consisted of a randomized trial with an active control group and thus offered better control for general and specific factors such as group and expectation and test–retest effects. In addition, Van der Gucht et al. only demonstrated effects over positive ED comparing pre-intervention and the follow-up measurements (4 months after finishing the MBSR), suggesting that those effects may take a longer time, and thus, our study might have failed to detect them. Finally, Van der Gucht et al.’s study used Ecological Momentary Assessment (EMA) while our study employed a self-reported questionnaire, resulting in differences in the assessment methodology. Therefore, a number of methodological reasons might explain the differences between the studies. Compared to the READ group, the MBSR group specifically improved negative ED as the program explicitly trains individuals to deliberately turn toward and explore unpleasant sensations and negative emotions (Guendelman et al., 2017). Moreover, it is possible that the negativity bias—the tendency to give more weight to negative experiences—may have played a role in biasing individuals toward focusing primarily on negative emotions, therefore specifically influencing the negative ED learning process.

Our study is in line with previous models that suggest that MBIs can enhance top-down mechanisms, such as attention control, cognitive reappraisal, and affect labeling, among others (Chiesa et al., 2013; Tang et al., 2015; Guendelman et al., 2017; Raugh and Strauss, 2024; for RCT studies: Goldin et al., 2021; Guendelman et al., 2022; Tang et al., 2016).

Exploratory analyses revealed, however, no significant associations between changes in ED and mental-health outcome variables, which could be due to a lack of statistical power as effect sizes for this type of association might be rather small.

Furthermore, it seems likely that multiple factors in addition to ED are contributing to mental health, and complex interactions with moderating and mediating factors might be at work; thus, it might be difficult to identify single contributions.

### Effects of MBSR on heart rate variability

Despite the fact that some studies have shown that MBIs could enhance cardiovascular recovery (Crosswell et al., 2017) and resting HRV levels longitudinally (Brown et al., 2021), our study could not show MBSR-related increases in resting HRV. Our findings are in line, however, with latest large studies (Lumma et al., 2015) and meta-analytical evidence (Brown et al., 2021), indicating that MBIs might not modify resting HRV. Nevertheless, this lack of modification could be due to the data collection environment as resting HRV was acquired during the brain scan MRI assessments. Therefore, our findings did not provide evidence for previous models suggesting that MBIs can enhance bottom-up mechanisms, such as interoceptive and bodily related processes (Chiesa et al., 2013; Guendelman et al., 2017).

There are different methodological approaches to investigate mindfulness, conceiving it as a current ‘mental state’ (during ongoing mindfulness meditation), an individual disposition or trait (dispositional mindfulness), or properly structured group interventions (MBIs) (Davidson, 2010). In a study investigating state HRV while practicing mindfulness meditation, authors could not demonstrate HRV increases during active meditation, indicating that mindfulness practice may recruit (conflicting) cognitive resources, such as in any active cognitive task, and may increase sympathetic rather than parasympathetic activation (Luque-Casado et al., 2016). In this context, MBSR may exert a conflicting effect over HRV mechanisms: on the one hand, increasing cognitive resources and sympathetic-related processes and, on the other hand, decreasing stress and arousal, thus increasing parasympathetic-related processes. Consequently, HRV might not be a good estimate for measuring mindfulness-related outcome effects.

Although previous literature has shown that resting HRV might be a reliable marker of stress and arousal processing abilities, reflecting the autonomic availability for stress processing (Balzarotti et al., 2017), it may index state availability of autonomic activation rather than trait dispositions for emotion-related bottom-up regulation. Future studies aiming to investigate bottom-up processes in the context of MBIs should focus on neuro-physiological markers that directly index these processes (i.e., insula activation), in the context of tasks where top-down processes can be controlled and disentangled (i.e., using signal detection manipulations) (i.e., Mylius et al., 2024).

### Limitations

Our study exhibits both strengths and limitations. Our method for estimating emotion differentiation, compared to EMA, might be less ecological and sensitive to detect changes over time or trajectories. Furthermore, there might be certain confounding factors, such as pre-intervention alexithymia or mindfulness levels, that might influence individuals’ capacity for changing emotion differentiation over time. Regarding HRV measurements, data collection occurred during the MRI brain scan, which could have increased participants’ stress levels, compared to other studies where heart rate data collection occurred in less stressful settings. Mental health outcomes were assessed using self-reported questionnaires, which are known to be susceptible to context effects and memory-related response biases. Furthermore, our study faced challenges in recruiting male participants, with female participants comprising 83% of our sample. This finding aligns with naturalistic studies showing greater female participation in meditation programs. Future investigations should implement better controls for gender balance, given its influence on emotion regulation capacities.

## Conclusion

Our study demonstrates, for the first time in an RCT, that MBIs can enhance negative emotion differentiation, while not increasing resting HRV, thus shedding light on the underlying emotion regulation-related processes.

## Data Availability

The raw data supporting the conclusions of this article will be made available by the authors without undue reservation.
