# Supplementary material for: Effects of a mindfulness intervention on emotion differentiation and heart rate variability
Source: Front Hum Neurosci. 2025 Jul 2;19:1515334. doi: 10.3389/fnhum.2025.1515334 (PMC12263662; doi:10.3389/fnhum.2025.1515334)
Supplement: Supplementary file 1 [file Table_1.docx]

**Table 1s.**

Demographics characteristics at baseline for subjects randomized to MBSR versus READ

|  | **MBSR**  *n = 30* | **READ**  *n= 29* | **t-test or χ2** |
| --- | --- | --- | --- |
| Females, n (%) | 25 (83%) | 24 (83%) | χ2*_1_* = 0.00 |
| Age, mean years (SD) | 40.2 (10.8) | 36.8 (10) | t*_60_* = 1.31 |
| After School Education, mean years (SD) | 6.20 (2.46) | 6.39 (2.61) | t*_60_* = 0.27 |

*SD, standard deviation; n = sample size; t-test = independent sample t test; χ2 = chi square.*

Eligibility and Inclusion criteria:

| Ages Eligible for Study: | 18 Years to 55 Years   (Adult) |
| --- | --- |
| Sexes Eligible for Study: | All |
| Accepts Healthy Volunteers: | Yes |

Inclusion Criteria:

- Healthy volunteers: Men or women
- Right-handed
- Normal or corrected-to-normal vision
- German as a first language
- No history of neurological or psychiatric problems, or use of controlled medication that might interfere with emotion or attention.
- No symptoms of claustrophobia
- Written informed consent form to participate in the study and neuro-imaging experiment.

Exclusion Criteria:

- Chemical dependence, including nicotine and alcohol
- Dementia or psychotic condition.
- Depression or use of antidepressants
- Pregnant or breast-feeding women
- Prior MBSR class or regularly practicing mindfulness meditation (or yoga) for three or more months.
- Non-controlled severe medical disease that might interfere with the performance of the study, such as neoplasms, cardiopathies, digestive pathologies, diabetes mellitus type I or type II
- Neoplasms in the central nervous system
- Tremor or dystonia in cephalic segment that hinders the performance of the MRI study (tremor equal to or higher than 3 in each corporal segment, according to the UPDRS scale)
- Fulfillment of any criterion of contraindication for the MRI exam (for instance, metallic implants, claustrophobia, pregnancy, use of pacemaker; intracranial aneurism clip; cochlear implants).
- Presence of odontological devices that might disturb the magnetic field or any sources of variation of the magnetic susceptibility
- Any other conditions the investigator might deem problematic for the inclusion of the volunteer in a trial of this nature will also be considered.
